# Identification of DGUOK-AS1 as a Prognostic Factor in Breast Cancer by Bioinformatics Analysis

**DOI:** 10.3389/fonc.2020.01092

**Published:** 2020-07-17

**Authors:** Yalun Li, Yiran Liang, Tingting Ma, Qifeng Yang

**Affiliations:** ^1^Department of Breast Surgery, Qilu Hospital, Cheeloo College of Medicine, Shandong University, Jinan, China; ^2^Department of Breast Surgery, The Affiliated Yantai Yuhuangding Hospital of Qingdao University, Yantai, China; ^3^Pathology Tissue Bank, Qilu Hospital of Shandong University, Jinan, China

**Keywords:** breast cancer, lncRNA, competitive endogenous RNA, DGUOK-AS1, prognosis

## Abstract

**Background:** Significant developments have been made in breast cancer diagnosis and treatment, yet the prognosis remains unsatisfactory. Accumulating evidence indicates that long non-coding RNAs (lncRNAs) play pivotal roles in the development and progression of human tumors. However, the regulatory mechanisms and clinical significance of most lncRNAs in breast cancer remain poorly understood.

**Methods:** The lncRNA, miRNA, and mRNA expression profiles were obtained from the Gene Expression Omnibus (GEO) and The Cancer Genome Atlas (TCGA) databases. A lncRNA-miRNA-mRNA regulatory network was constructed and visualized using Cytoscape. The protein-protein interaction (PPI) network was constructed using the STRING database and hub genes were extracted using the cytoHubba plugin. Gene Ontology and Kyoto Encyclopedia of Gene and Genomes analyses identified the functions and signaling pathways associated with these differentially expressed mRNAs (DEmRNAs). Expression of the key lncRNA and the relationship with prognosis of patients with breast cancer were evaluated.

**Results:** Six differentially expressed lncRNAs (DElncRNAs), 29 differentially expressed miRNAs (DEmiRNAs), and 253 DEmRNAs were selected to construct the regulatory network. A PPI network was established and seven hub genes were identified. A lncRNA-miRNA-hub gene regulatory sub-network was established containing two DElncRNAs, five DEmiRNAs, and seven DEmRNAs. Hub genes were associated with breast cancer onset and progression. The upregulated DGUOK-AS1 was identified as the key lncRNA in breast cancer based on the competing endogenous RNA network. High DGUOK-AS1 expression was associated with adverse prognosis in patients with breast cancer and a prognostic nomogram built on Grade, LN status, and DGUOK-AS1 expression shows significant prognostic value.

**Conclusions:** Our results reveal the significant roles of lncRNA/miRNA/mRNA regulatory networks in breast cancer and identified a novel prognosis predictor and promising therapeutic target for patients with breast cancer.

## Introduction

Worldwide, breast cancer (BC) has a high level of morbidity. In 2018, BC was responsible for ~2.1 million new cases and 627,000 deaths, making it the most frequently diagnosed malignancy and the second leading cause of cancer-related death among women (1). Although there has been significant progress in personalizing treatment for BC, the 5-year overall survival remains relatively poor due to tumor heterogeneity. Therefore, elucidation of the molecular mechanisms underlying BC occurrence and progression is essential to enable the identification of novel therapeutic targets and potential molecular biomarkers for prognosis prediction.

Long non-coding RNAs (lncRNAs) are transcripts >200 nucleotides in length lacking protein-coding potential (2). LncRNAs play essential roles in regulating various biological processes, including cell cycle control, drug resistance, and tumorigenesis (3). Recent discoveries have indicated that several lncRNAs are frequently dysregulated in breast cancer and play a central role in tumorigenesis and metastasis by regulating gene expression (4, 5), indicating their diagnostic and prognostic value.

LncRNAs can function as miRNA sponges to cross-talk with mRNAs based on the competing endogenous RNA (ceRNA) hypothesis (6). Increasing evidence suggest that a single lncRNA may contain multiple binding sites for various miRNAs thereby modulating several targeted mRNAs and creating a dynamic regulatory network, increasing the complexity of lncRNAs regulatory mechanism. Integrative analysis of the ceRNA network revealed various significant lncRNAs in primary open angle glaucoma, providing novel strategies for further functional studies of lncRNAs (7). Tao et al. constructed a ceRNA network and found a variety of functional lncRNAs with significant diagnostic and prognostic values for patients with dilated cardiomyopathy (8). Additionally, various potential gastric cancer prognostic lncRNAs were identified based on integrated ceRNA network analysis (9). Moreover, Liu et al. identified several prognostic markers for glioblastoma based on lncRNA-related ceRNA network analysis (10). Therefore, ceRNA network construction will provide a comprehensive view of lncRNA-associated crosstalk, aid our understanding of cancer development and progression, and facilitate the identification of novel molecules for cancer diagnosis and treatment.

**Figure 1:**
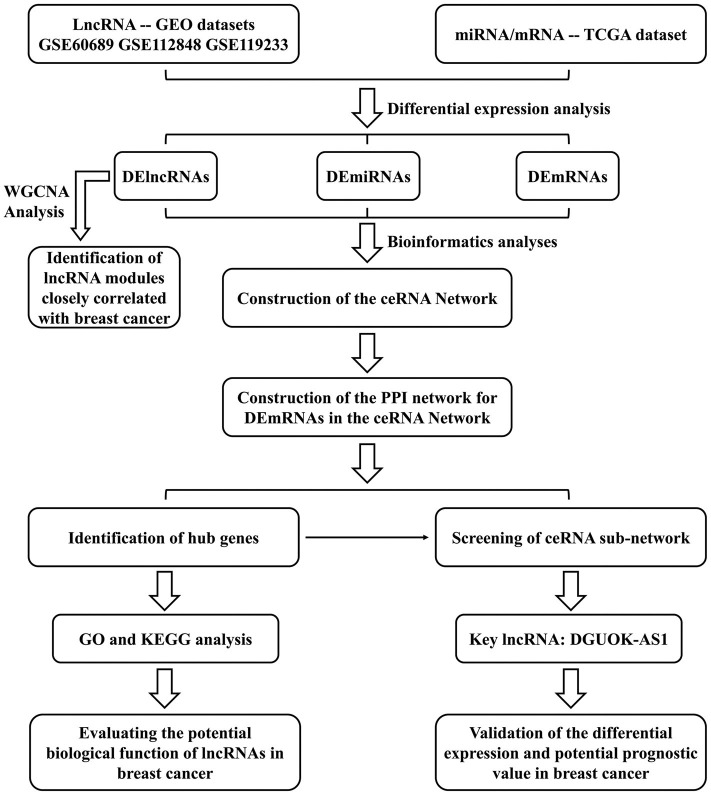
Flowchart of the approaches used in this study to construct the lncRNA-based prognostic risk score model.

We constructed a BC lncRNA-miRNA-mRNA ceRNA network based on data obtained from the GEO and TCGA databases using computational, experimental, and bioinformatic methods and bioinformatic tools. A protein-protein interaction (PPI) network was constructed using the Search Tool for the Retrieval of Interacting Genes database and the hub genes were extracted using the cytoHubba plugin. Gene Ontology (GO) and Kyoto Encyclopedia of Gene and Genomes (KEGG) analyses were used to identify the functions and signaling pathways associated with hub genes, indicating the potential mechanisms of lncRNAs in the occurrence and development of BC. Finally, based on ceRNA theory, the lncRNA deoxyguanosine kinase antisense RNA 1 (DGUOK-AS1)-centric subnetwork, based on the hub genes, was constructed. We concentrated on the comprehensive analysis of DGUOK-AS1, which was upregulated in the three datasets and regulated the expression of several sub-network hub genes. DGUOK-AS1 overexpression was further validated in a cohort of BC tissues and cells. Using Cox and Lasso analyses, we found that high DGUOK-AS1 expression level was correlated with poor prognosis in patients with BC. The study design flowchart is shown in Figure 1. These results provide insight into the roles of the ceRNA network in cancer and revealed the prognostic value of DGUOK-AS1 in BC. Moreover, DGUOK-AS1 might be a potential biomarker for prognosis prediction in BC.

## Materials and Methods

### Clinical Specimens

Patients with BC undergoing surgery at the Qilu Hospital of Shandong university between July 2008 and January 2015 (*n* = 182) were included. Normal and tumor tissues were stored at −80°C until RNA extraction. Detailed clinical and pathological information are listed in Table S1. Written informed consent was obtained from all patients and all experimental procedures were approved by the Ethical Committee of Qilu Hospital of Shandong University.

### Dataset Collection

Microarray data used were obtained from the GEO database (http://www.ncbi.nlm.nih.gov/gds/) (11). Three BC-related datasets were analyzed, including GSE60689 (two pairs of primary BC tissue and normal tissues), GSE112848 (three pairs of primary BC tissue and paracancerous tissues), and GSE119233 (twenty primary BC tissues and ten normal tissues). mRNA (1072 BC tissues and 99 normal tissues) and miRNA (1066 BC tissues and 90 normal tissues) expression data were acquired from TCGA (https://portal.gdc.cancer.gov/).

### Identification of Differentially Expressed Genes

The Limma package of R software was used to identify differentially expressed lncRNAs (DElncRNAs), while the edgeR package was used to identify differentially expressed miRNAs (DEmiRNAs) and mRNAs (DEmRNAs) between normal and tumor samples. The thresholds for selection of differentially expressed lncRNAs were *P* < 0.01 and |log2 fold change (FC)| > 2, while the cut-off values of miRNA and mRNA were set at *P* < 0.01 and |log2 FC| > 1.

### ceRNA Network Construction

The DElncRNAs common among the three GEO databases were selected for further investigation. lncRNA-miRNA interactions were predicted using DIANA-LncBase v2 (12).

To maximize data reliability, target miRNAs were further screened using the DEmiRNAs obtained from the TCGA database. miRNA-mRNA interactions were predicted using the miRTarBase (http://mirtarbase.mbc.nctu.edu.tw/) (13), Starbase (http://starbase.sysu.edu.cn/index.php) (14), TargetScan (http://www.targetscan.org) (15), and miRDB (http://www.mirdb.org/) databases (16). mRNAs recognized by more than three databases were considered candidate targets. Then, they were intersected with the identified DEmRNAs to get the final functional DEmRNAs targeted by the DEmiRNAs. A lncRNA-associated regulatory network was established and visualized using Cytoscape software (version 3.6.1) (17).

### Construction and Analysis of the Protein-Protein Interaction (PPI) Network

A total of 253 DEmRNAs were obtained and imported to the Search Tool for the Retrieval of Interacting Genes (http://string-db.org/) database to evaluate protein-protein interaction (18). The database provides a comprehensive score for each protein-protein association distributed between 0 and 1; the higher the interaction score, the more reliable the relationship. Consistent with other research, an interaction score > 0.6 was used as the cut-off criterion. Then, Cytoscape software was used to visualized the PPI network. The cytoHubba plugin of Cytoscape software, a widely used plugin for identifying important nodes and subnetworks, was used to identify the hub genes in the PPI network (19).

### Functional Enrichment Analysis

To better understand the underlying function of potential targets, the hub genes were analyzed using functional enrichment analysis. GO is a widely-used tool for annotating genes with functions (20), including molecular function, biological pathways, and cellular components. KEGG is a resource for understanding high-level gene functions (21), including molecular interactions, and reaction and relation networks. ClusterProfiler package in R was employed to obtain the enriched GO terms and KEGG pathways. “*P* < 0.05” was set as the inclusion criterion.

### Cell Culture

Cell lines were purchased from the American Type Culture Collection (ATCC, USA). MCF10A cells were cultured in DMEM/F12 (Invitrogen, USA), containing 5% horse serum (Invitrogen, USA), 10 μg/ml insulin, 20 ng/ml (epidermal growth factor) EGF, 100 ng/ml cholera toxin, 0.5 μg/ml hydrocortisone, 100 U/ml penicillin, and 100 μg/ml streptomycin. MCF-7, MDA-MB-453, MDA-MB-468, and MDA-MB-231 cells were routinely cultured in DMEM (Invitrogen, USA). T47D, ZR75-1, and SKBR3 cells were cultured in RPMI 1640 medium. The medium was supplemented with 10% fetal bovine serum (Hyclone), 100 U/ml penicillin, and 100 μg/ml streptomycin. The medium for T47D cells also contained 10 μg/ml insulin. Cells were cultured in a humidified atmosphere with 5% CO_2_ at 37°C.

### RNA Extraction and qRT-PCR

Total RNA was extracted from tissues or cells using Trizol reagent (Invitrogen, USA) following the manufacturer's instructions. Then, mRNA was reverse transcribed using the PrimeScript reverse transcriptase reagent kit (Takara, Shiga, Japan). β-actin was used as the endogenous control for DGUOK-AS1, and U6 was used as the endogenous control for hsa-miR-497-5p. DGUOK-AS1 and hsa-miR-497-5p expression was calculated using the 2^−ΔΔCT^ method. Primers used are listed in Table S2.

### Weighted Gene Co-expression Network Analysis (WGCNA) Analysis

WGCNA can detect lncRNA modules and evaluate the correlation of each module with the characteristics of the samples (22). We integrated and batched the data from GSE60689, GSE112848, and GSE119233 to perform WGCNA Analysis. The WGCNA package of R software was applied to construct traits-related modules. The soft-thresholding power (β value) was set as 10, so that the scale-free topology fit index (scale-free R2) was >0.8 and maintained optimal mean connectivity. Then, the adjacency matrix, including 40 samples, was transformed into a topological overlap matrix to evaluate the lncRNA co-expression similarity. LncRNA hierarchical clustering was created based on the topological overlap matrix dissimilarity, dividing the lncRNAs into different modules. LncRNAs with high absolute correlation were clustered into the same module. The dissimilarity of the module eigengenes was calculated using the moduleEigengenes function in the R WGCNA package. Finally, the correlation between module eigengenes and clinical traits was determined using Pearson's correlation test and *P* < 0.05 was considered significant.

### Statistical Analyses

Statistical analyses were performed using SPSS 21.0 (Chicago, IL, USA) and R software. Data are expressed as mean ± standard deviation of three independent experiments. The *P* < 0.05 was considered statistically significant. The student's *t*-test was used to analyze differences between groups. Survival analysis was determined using Kaplan-Meier curves and the log-rank test was used to evaluate the significance. The correlation between lncRNA expression and clinical characteristics was assessed by chi-square test. Uni-variate cox regression analysis, lasso regression analysis, multi-variate cox regression analysis, and receiver operating characteristic curve (ROC) analysis were conducted in R software to evaluate the prognostic potential of the risk factors in patients with BC.

## Results

### Identification of Differentially Expressed Genes in Breast Cancer

**Figure 2:**
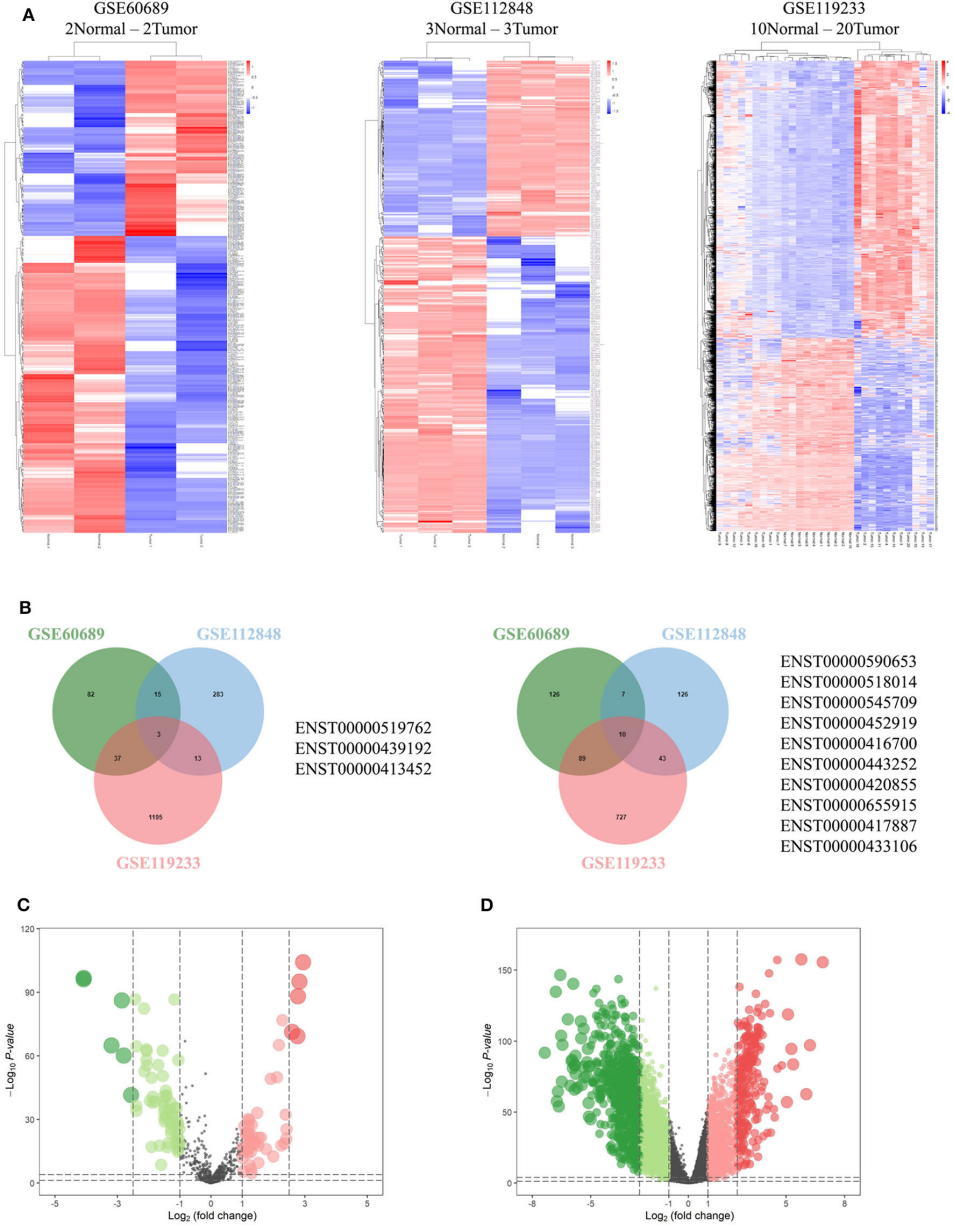
Differentially expressed genes. **(A)** Heatmaps of GSE60689, GSE112848, and GSE119233 showing the differentially expressed lncRNAs (DElncRNAs). **(B)** Comparison of the differentially over-expressed or down-regulated lncRNAs in the three gene expression omnibus datasets. **(C,D)** Volcano plot of differentially expressed microRNAs **(C)** and mRNAs **(D)** from The Cancer Genome Atlas. Normalized expression levels are shown in descending order from green to red.

Integrated analysis of GSE60689, GSE112848, and GSE119233 datasets using the R project limma package (*P* < 0.01 and |log2FC| > 2.0) identified 369, 500, and 2,617 differentially expressed lncRNAs (DElncRNAs), respectively. GSE60689 included 137 upregulated and 232 downregulated lncRNAs, GSE112848 had 314 upregulated and 186 downregulated lncRNAs, and GSE119233 had 1248 upregulated and 869 downregulated lncRNAs (Figure 2A). Then, we took the intersection of DElncRNAs of the three datasets, identifying three commonly upregulated lncRNAs and 10 commonly downregulated lncRNAs (Figure 2B, Table S3). The TCGA data were analyzed using the “edgeR” package in R (*P* < 0.01 and |log2 (FC)| > 1.0). A total of 112 differentially expressed miRNAs were obtained, 66 of which were downregulated and 46 of which were upregulated (Figure 2C). Moreover, the same analysis of the TCGA data revealed 4,328 differentially expressed mRNAs including 1,638 upregulated mRNAs and 2,690 downregulated mRNAs (Figure 2D).

### WGCNA Analysis Revealed Hub Modules Closely Correlated With BC

**Figure 3:**
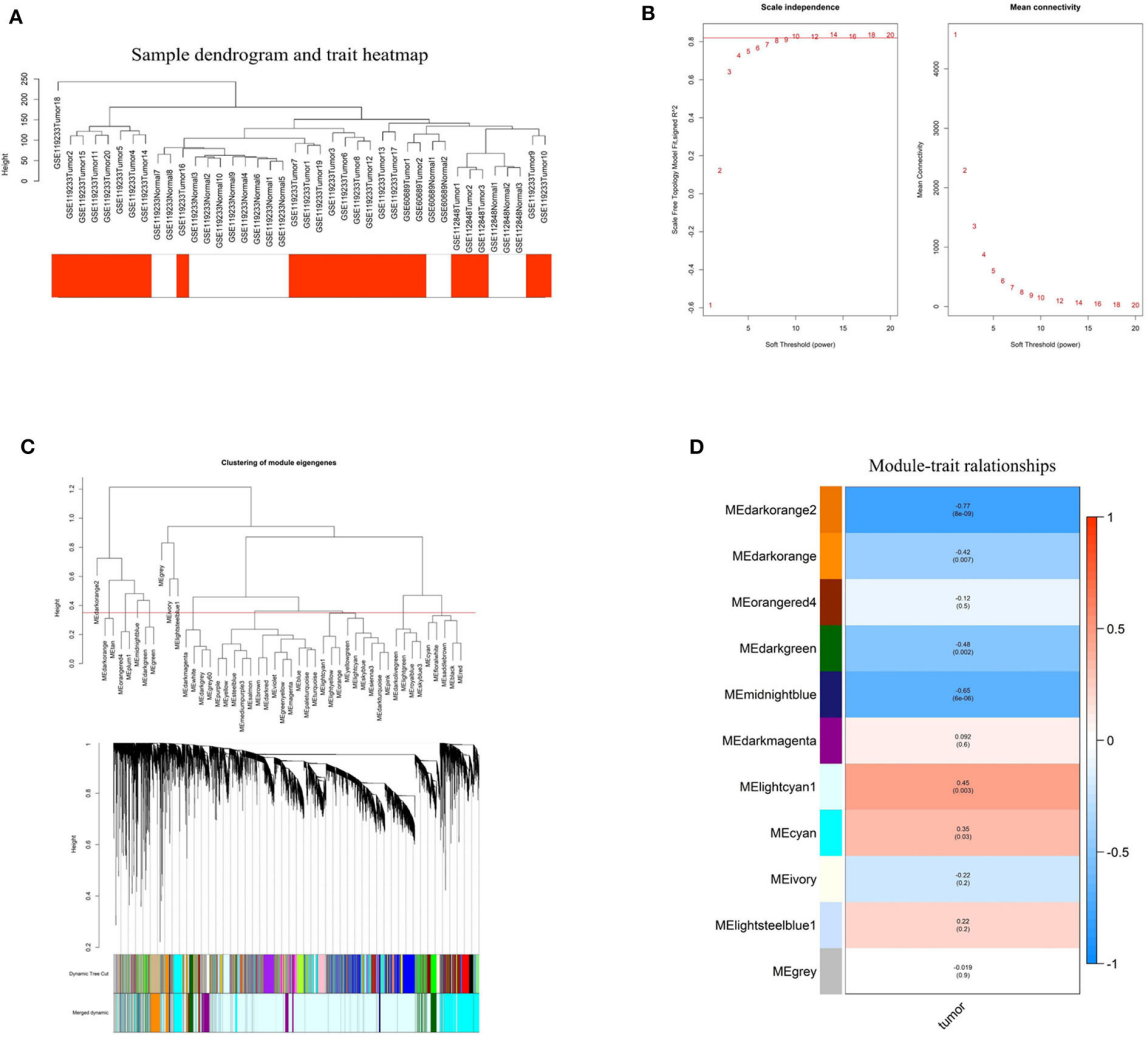
Identification of hub modules correlated with breast cancer using weighted gene co-expression network analysis. **(A)** Sample dendrogram and trait heatmap based on the combined data from GSE60689, GSE112848, and GSE119233. **(B)** Analyses of the Scale-Free Topology Model Fit and the Mean Connectivity for different soft threshold powers. **(C)** Clustering dendrograms of lncRNAs based on combined data from GSE60689, GSE112848, and GSE119233. **(D)** The correlation between various modules and tumor tissues. The correlation coefficient and *P*-value are presented in the heatmap.

To investigate the relationship between differentially expressed lncRNAs and BC, WGCNA analysis was performed. The sample sizes of each dataset were too small to perform WGCNA analysis individually. Therefore, the three datasets were combined using batch normalization in R project. The data matrix included 40 samples and the best soft threshold (power) was 10 (Figures 3A,B). After analyzing and classifying all lncRNAs, 11 modules were identified (Figures 3C,D). The heatmap of module-trait relationships revealed that the lightcyan1 module (*P* = 0.003, correlation coefficient = 0.45) and cyan module (*P* = 0.03, correlation coefficient = 0.35) were positively associated with tumor tissues, and the darkorange2 module (*P* = 8e-09, correlation coefficient = −0.77), midnightblue module (*P* = 6e-06, correlation coefficient = −0.65), darkgreen module (*P* = 0.002, correlation coefficient = −0.48), and darkorange module (*P* = 0.007, correlation coefficient = −0.42) were negatively associated with tumor tissues. Significantly, the upregulated ENST00000519762 belongs to the lightcyan1 module, and ENST00000439192 and ENST00000413452 belong to the cyan module (Table S4). Moreover, most downregulated DElncRNAs belongs to the darkorange module (Table S4). These results highlight the pivotal role of DElncRNAs in BC.

### Construction of the ceRNA Network

**Figure 4:**
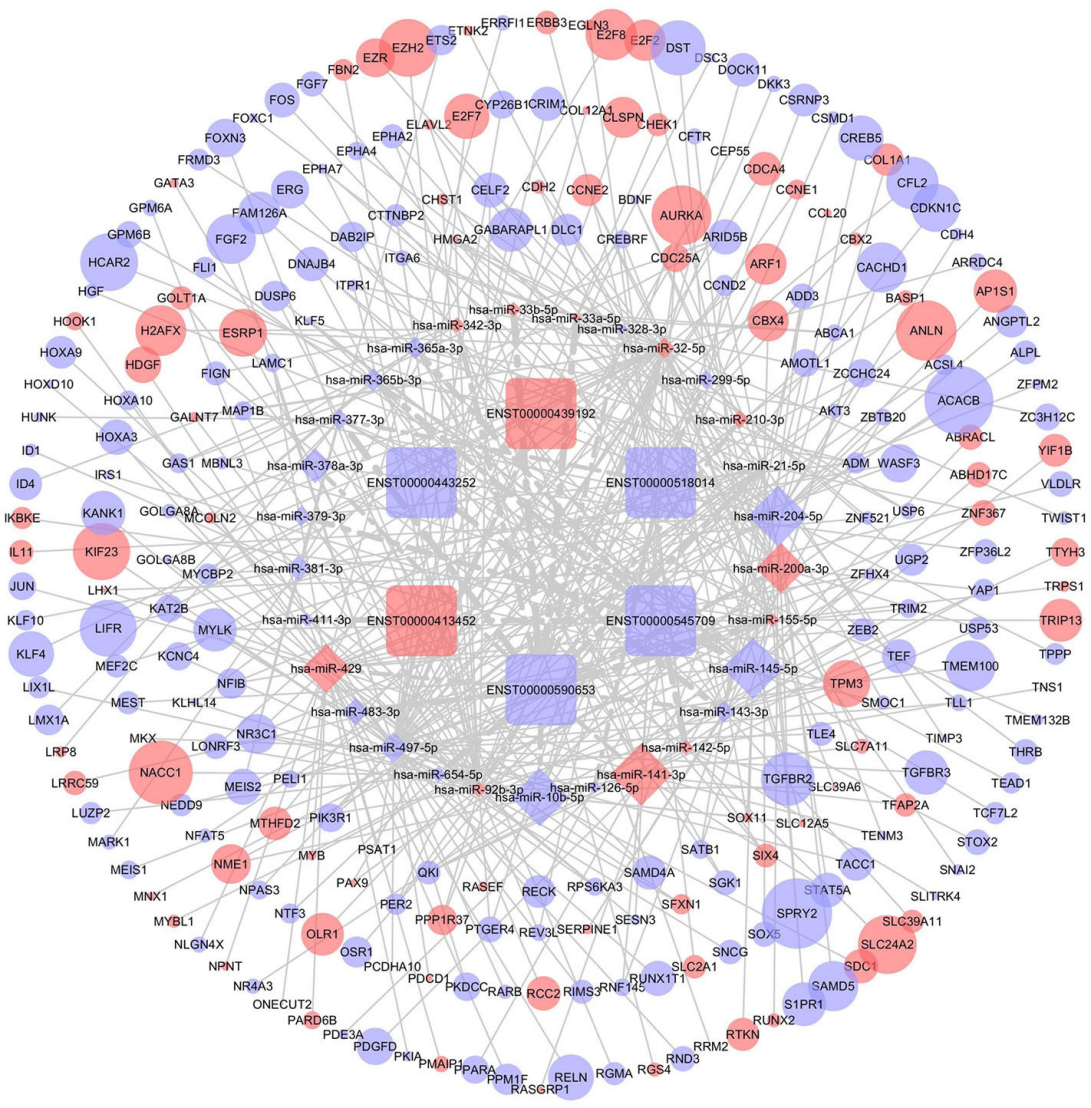
The competing endogenous RNA (ceRNA) network of lncRNA (lncRNA)-microRNA (miRNA)-mRNA in breast cancer. Rectangles represent lncRNAs, diamonds represent miRNAs, ellipses represent mRNAs, and gray lines represent lncRNA-miRNA-mRNA interactions. Red nodes indicate upregulated expression and purple nodes denote downregulated expression. The node size is dependent on the log2 (FC) of the genes.

DIANA-LncBase v2 was used to predict the miRNAs targeted by 13 lncRNAs and, after removal of duplicates, 1,028 miRNAs were obtained. After crosschecking with the DEmiRNAs, only 57 common miRNAs and 77 lncRNA-miRNA pairs remained. We identified mRNAs targeted by these DEmiRNAs using the miRTarBase, Starbase, TargetScan, and miRDB databases. After intersecting the predicted mRNAs with the previously identified DEmRNAs, 253 DEmRNAs were obtained. Finally, a lncRNA-miRNA-mRNA network, including six lncRNAs, 29 miRNAs, and 253 mRNAs, was constructed and visualized using Cytoscape (Figure 4).

### Construction of the PPI Network

**Figure 5:**
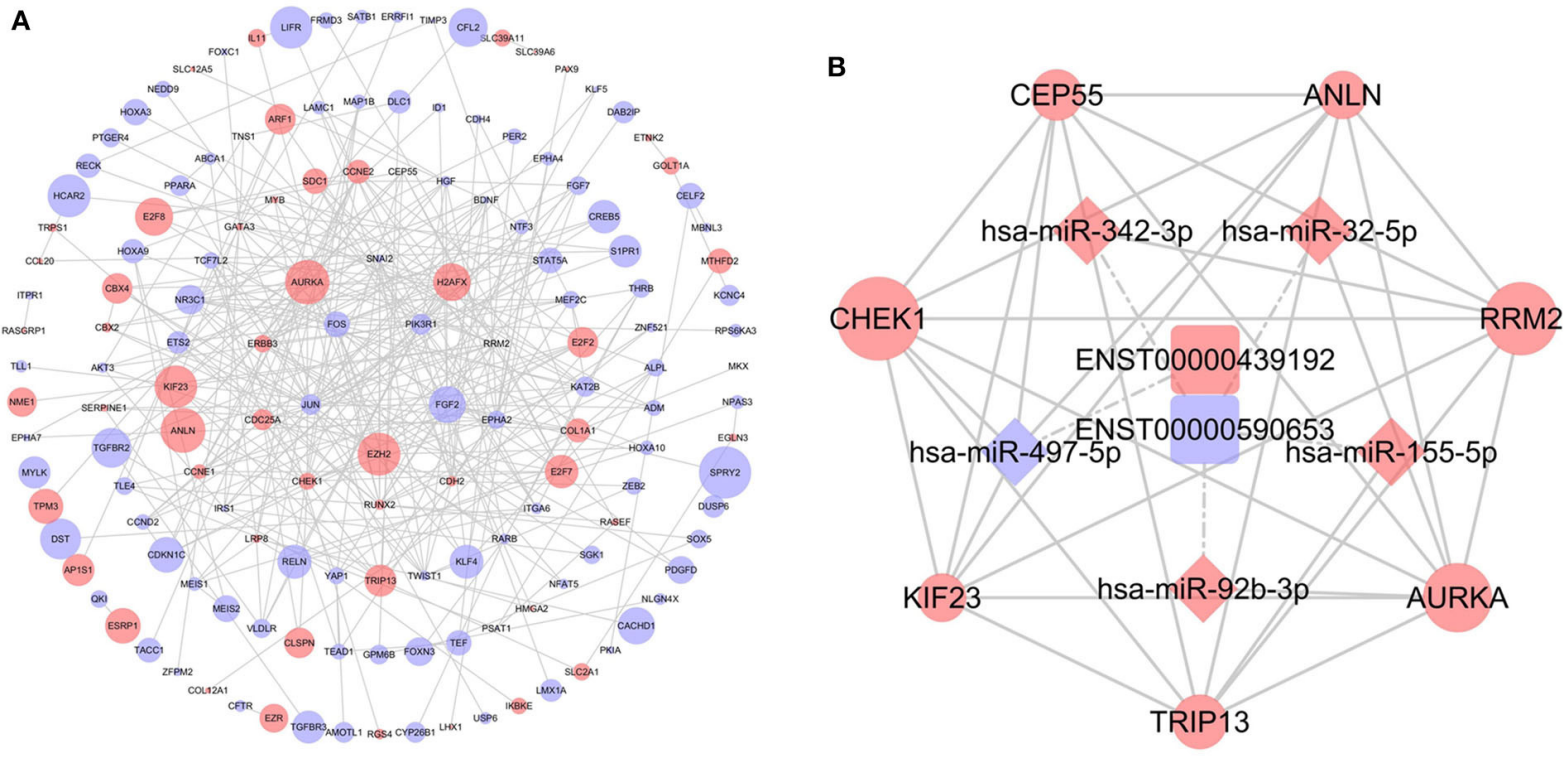
Identification of hub genes from the protein-protein interaction (PPI) network. **(A)** PPI network diagram of 253 genes, consisting of 150 nodes and 331 edges. **(B)** The regulatory network based on the top 10 hub genes.

To further determine the biological function of potential lncRNA-related genes, we constructed a PPI network using the 253 DEmRNAs (Figure 5A). After removing unconnected nodes, the PPI network contained 150 nodes and 331 edges. The hub genes in the network were identified using the cytoHubba plugin of Cytoscape. According to cytoHubba's MCC ranking, the top hub genes were *CHEK1, CEP55, ANLN, RRM2, AURKA, TRIP13*, and *KIF23*. Then, a lncRNA-miRNA-hub gene network was constructed (Figure 5B), including two lncRNAs (ENST00000439192 and ENST00000590653), five miRNAs (hsa-miR-497-5p, hsa-miR-342-3p, hsa-miR-32-5p, hsa-miR-155-5p, and hsa-miR-92b-3p), and the hub genes.

### GO and KEGG Analysis to Predict Probable Hub Gene Functions

**Figure 6:**
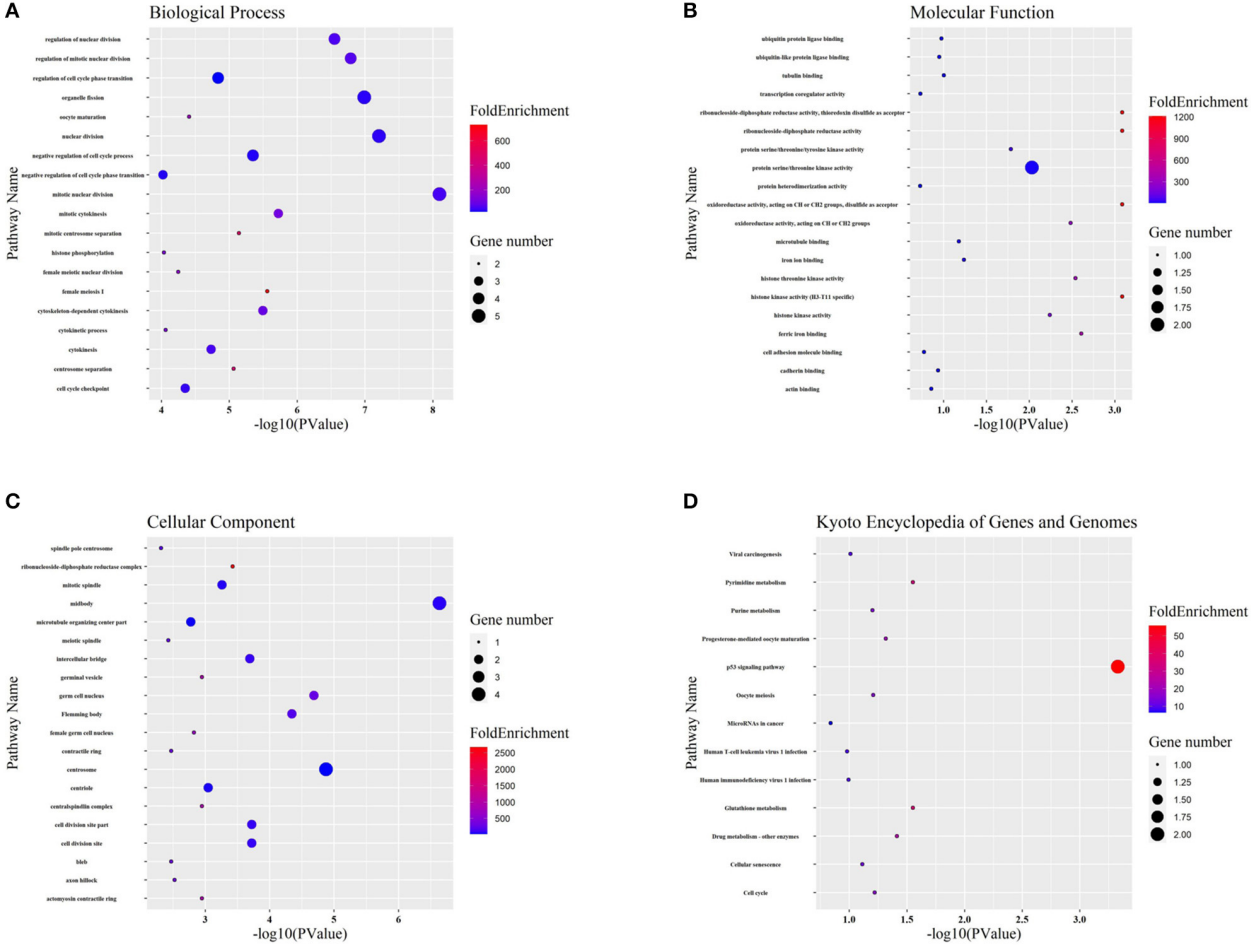
GO and KEGG functional analyses. **(A-C)** Gene ontology (GO) analysis of hub genes. The biological process (BP), molecular function (MF), and cellular component (CC) of potential targets were clustered based on ClusterProfiler package in R software. The X-axis reflects the gene counts and the Y-axis shows the enriched pathways. **(D)** The enriched KEGG signaling pathways were selected to demonstrate the primary biological actions of major potential targets. The X-axis indicates the gene ratio and the enriched pathways are presented in the Y-axis.

Targeted genes might shed light on the functions of related lncRNAs. Therefore, GO enrichment and KEGG pathway analyses were performed to explore hub gene functions. In terms of biological process (Figure 6A), hub genes were mainly associated with mitosis and the cell cycle. In terms of molecular function (Figure 6B), hub genes were mostly enriched in protein kinase activity, iron binding, and protein binding. The most enriched cellular component terms (Figure 6C) were midbody, centrosome, and spindle. In addition, the most enriched KEGG pathway terms (Figure 6D) for the hub genes were the p53 signaling pathway, pyrimidine metabolism, glutathione metabolism, and drug metabolism.

### Identification of DGUOK-AS1 Regulatory Network

**Figure 7:**
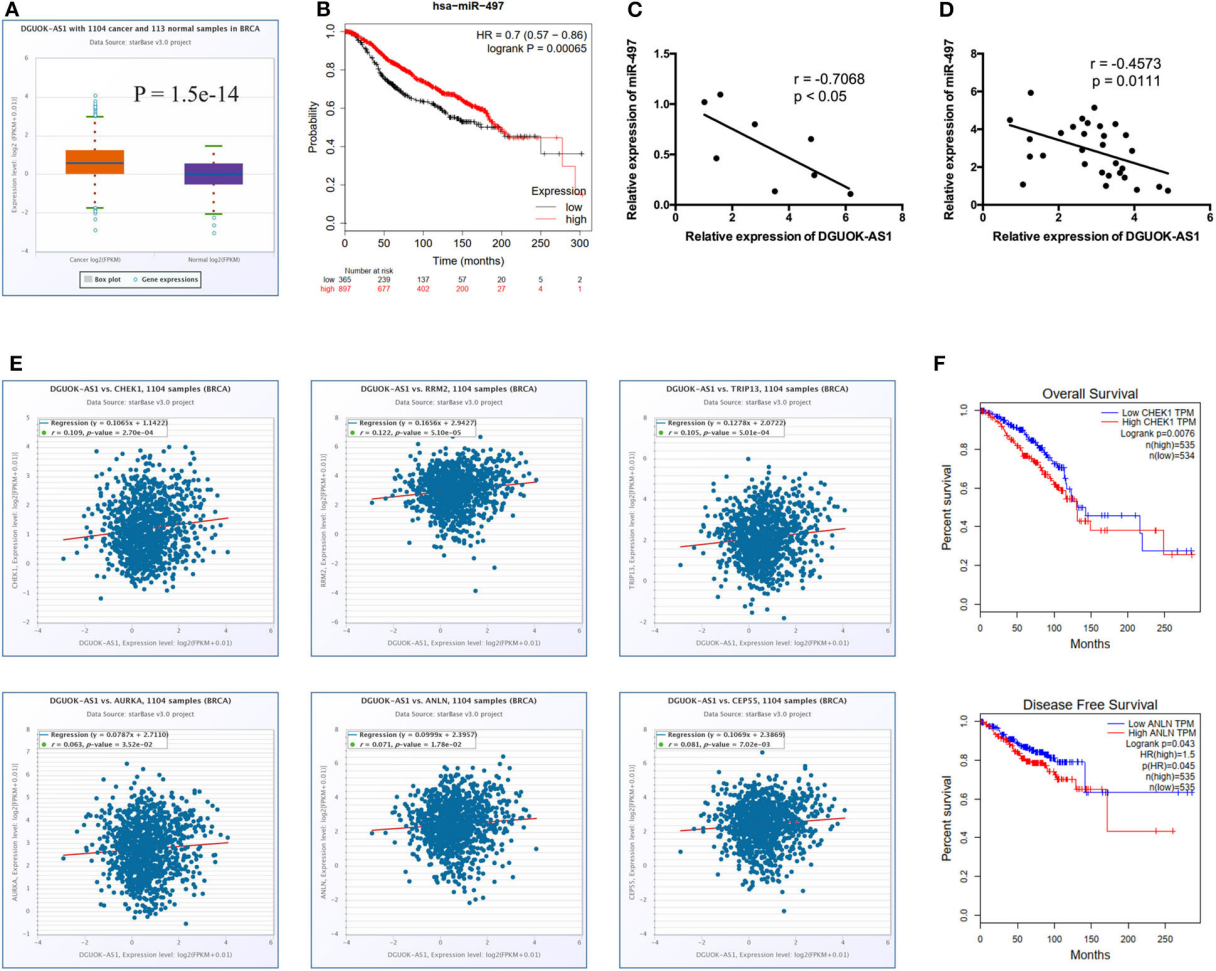
Identification of DGUOK-AS1 regulatory network. **(A)** The expression of DGUOK-AS1 in TCGA. **(B)** Overall survival of hsa-miR-497-5p using Kaplan-Meier Plotter. **(C,D)** The association between DGUOK-AS1 and hsa-miR-497-5p in breast cancer cells and breast cancer tissues. **(E)** The association between DGUOK-AS1 and hub genes using ENCORI database. **(F)** Overall survival of CHEK1 and disease-free survival of ANLN using GEPIA database.

Significantly, all hub genes were increased in BC tissues compared to normal tissues. Based on the ceRNA theory, the upregulated ENST00000439192 (also named DGUOK-AS1) was associated with one downregulated miRNA (hsa-miR-497-5p) and seven upregulated mRNAs (*CCNE1, CDC25A, CEP55, CHEK1, E2F7, KIF23*, and *AURKA*). Expression of DGUOK-AS1 was significantly upregulated in TCGA database BC tissues (Figure 7A). Using the Kaplan-Meier Plotter, we found that highly expressed miR-497-5p was positively associated with overall survival in patients with BC (Figure 7B). Moreover, we identified a negative correlation between DGUOK-AS1 and miR-497-5p in BC cells and tissues (Figures 7C,D). DGUOK-AS1 was positively associated with most hub genes (Figure 7E), further supporting the reliability of the regulatory network. Additionally, increased *CHEK1* expression was associated with poor overall survival in patients with BC and high *ANLN* expression was related to poor disease-free survival (Figure 7F). Therefore, this lncRNA-miRNA-hub gene network indicated that DGUOK-AS1 might be a major molecule associated with BC development and progression, capable of sponging miR-497-5p to regulate the expression of several hub genes.

### Validation of the Differential Expression and Potential Prognostic Value of DGUOK-AS1 in BC

**Figure 8:**
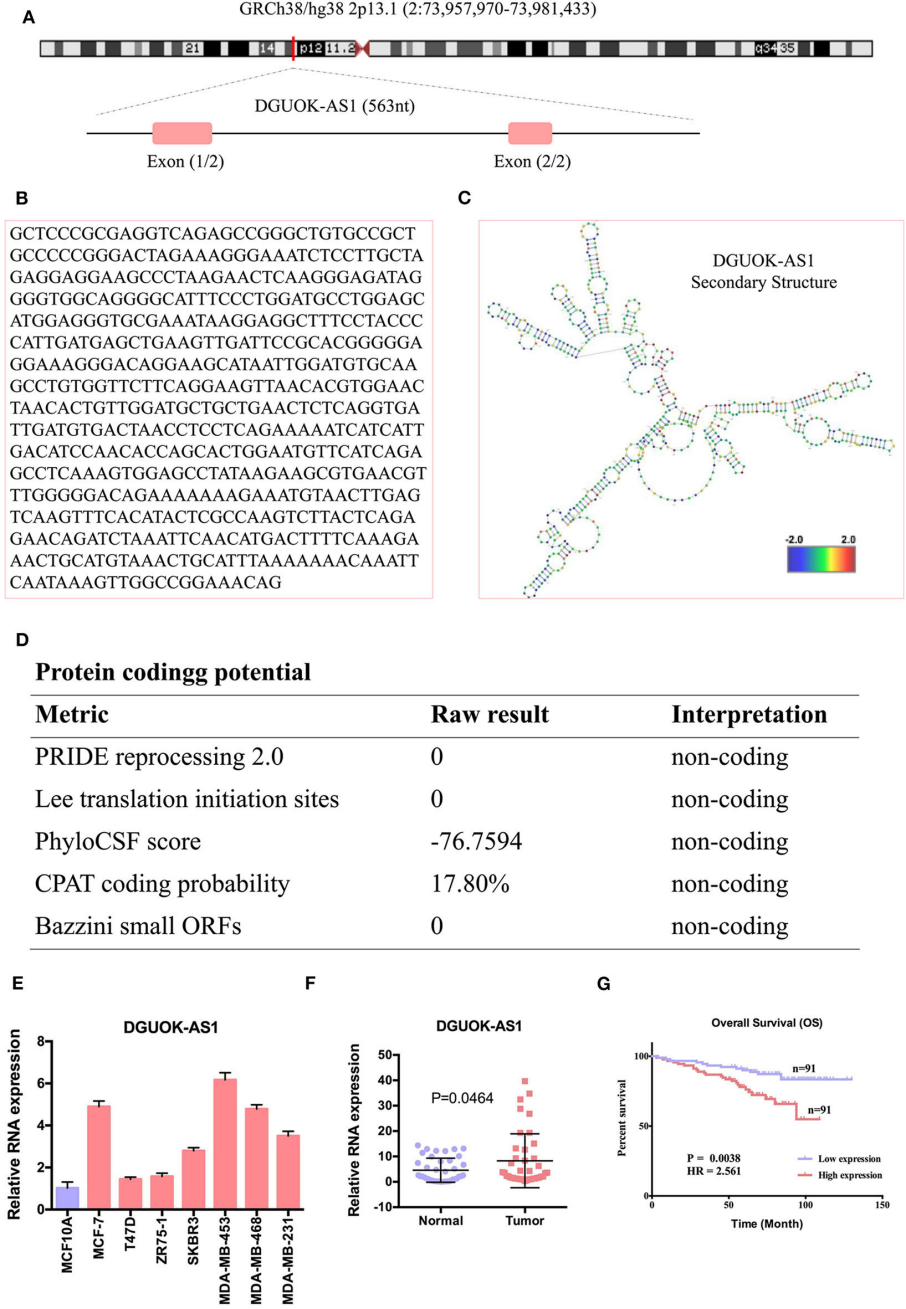
The characteristic of DGUOK-AS1. **(A)** Schematic diagram of the DGUOK-AS1 genomic locus in humans. Pink rectangles represent exons. **(B)** DGUOK-AS1 sequence. **(C)** DGUOK-AS1 secondary structure obtained from AnnoLnc (http://annolnc.cbi.pku.edu.cn/). **(D)** DGUOK-AS1 coding potential analysis. Five kinds of metrics were used and show that DGUOK-AS1 has no coding potential. **(E)** Differences in DGUOK-AS1 expression in breast cancer cells and normal cell. **(F)** Differences in DGUOK-AS1 expression in 40 breast cancer tissues and 40 normal tissues. β-actin was used as a control. **(G)** DGUOK-AS1 overall survival curve.

We selected the unannotated lncRNA, DGUOK-AS1, for further investigation. In human, DGUOK-AS1 is located on 2q24.32 and is composed of two exons with a full length of 563 nt (Figure 8A). DGUOK-AS1 sequence and secondary structure are shown in Figures 8B,C, and *in silico* analysis revealed that DGUOK-AS1 has little protein coding potential (Figure 8D). This was supported by the lack of a valid Kozak consensus sequence in DGUOK-AS1 (23). To further investigate the role of DGUOK-AS1 in BC, qRT-PCR was used to verify differential DGUOK-AS1 expression in BC cells (Figure 8E). Compared to that measured in normal breast cells (MCF10A), DGUOK-AS1 expression was significantly higher in BC cells (MCF-7, T47D, ZR75-1, SKBR3, MDA-MB-453, MDA-MB-468, and MDA-MB-231). Investigation of DGUOK-AS1 expression levels in 40 paired BC and normal breast tissues revealed that DGUOK-AS1 was significantly overexpressed in BC tissues compared with adjacent normal tissues (Figure 8F). To explore the potential prognostic value of DGUOK-AS1, a cohort of 182 patients with BC with detailed clinicopathologic information and survival data were included. Using the median DGUOK-AS1 expression in patients with BC as the threshold, patients were divided into high- and low-expression groups. The Kaplan-Meier survival curve revealed that higher DGUOK-AS1 expression was associated with poorer overall survival in patients with BC (Figure 8G). These results indicate that DGUOK-AS1 upregulation may be involved in the progression of BC.

### Confirmation of the Potential Prognostic Value of DGUOK-AS1 in Breast Cancer

**Figure 9:**
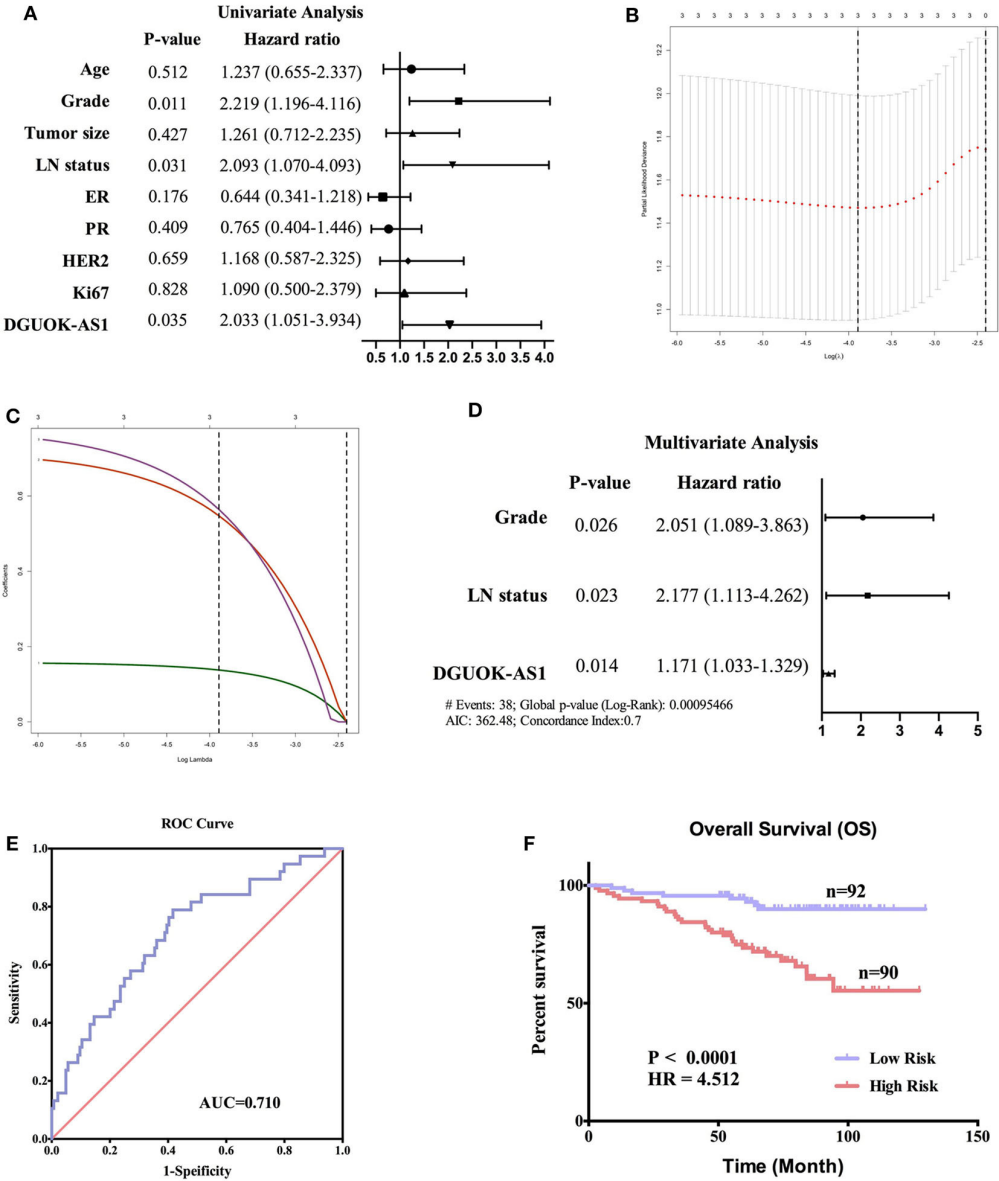
Cox regression analyses in 182 breast cancer patients. **(A)** Univariate analysis of nine risk factors in breast cancer patients. Those with *P* < 0.05 were selected for further study. **(B,C)** Lasso regression analysis of three risk factors. **(D)** Multivariate analysis of three risk factors. **(E)** The receiver operating characteristic curve of the risk score model. **(F)** Survival curves of the high- and low-risk groups in 182 breast cancer patients based on the risk score model.

To verify the prognostic value of DGUOK-AS1, univariate, lasso, and multivariate analyses were used to assess the risk factors in patients with BC. Univariate analysis revealed that Grade, LN status, and DGUOK-AS1 expression were significant risk factors in patients with BC (Figure 9A). This was consistent with the lasso analysis results (Figures 9B,C). Multivariate analysis conducted with all three risk factors indicated that the three variables were independent risk factors for overall survival and were selected to establish the prognostic model: risk Score = 0.718 * Grade + 0.778 ^*^ LN status + 0.158 * DGUOK-AS1 (Figure 9D). The C-index was 0.702 (95% CI = 0.616–1.560). ROC analysis revealed that the most optimal cutoff value for dividing patients with BC into high- and low-risk groups was 2.314 and the area under the ROC curve was 0.710 (Figure 9E). Based on this cutoff value, the 182 patients with BC were classified into two groups. The Kaplan-Meier survival curve revealed that the high-risk group was closely associated with poor survival status (Figure 9F), indicating that these three risk factors perform satisfactorily to predict prognosis in patients with BC.

## Discussion

Although advances in the diagnosis and treatment of BC have improved patient prognosis, BC remains incurable. A potential contributing factor is the high heterogeneity of BC cells that lead to disease recurrence and metastasis (24). It is important to comprehensively understand the molecular mechanisms involved in BC occurrence and development and to identify novel prognostic predictors. Recently, with the emergence of non-coding RNAs (25), researchers have shifted their investigation attention from traditional protein-coding genes to ncRNAs, especially lncRNAs. LncRNAs play significant roles in carcinogenesis and the progression of cancer through different regulatory mechanisms (26). Moreover, several lncRNAs were identified as diagnostic or prognostic biomarkers for cancers, and might be useful in individualized therapy to maximize efficacy, leading to reduced disease recurrence and metastasis. However, the clinical value of most lncRNAs in BC remains unclear. In this study, we identified several uncharacterized lncRNAs that target the regulation of downstream genes through sponging miRNAs. Functional analysis of target genes was performed to better understand the potential functions of lncRNA. A significantly upregulated lncRNA, DGUOK-AS1, was selected to explore its role in prognosis prediction in patients with BC.

To identify lncRNAs that are dysregulated in BC, the publicly available GEO database was explored and three GPL16956 datasets were analyzed. These analyses identified 13 BC-related lncRNAs. Various studies have reported that lncRNAs could serve as ceRNAs, protecting target mRNAs from repression (27). To elucidate the role of the lncRNA-related ceRNA network in BC development and progression we analyzed miRNA and mRNA expression using the TCGA database, and identified 112 dysregulated miRNAs and 4,328 dysregulated mRNAs. Combining these results with website prediction, a BC-related lncRNA-miRNA-mRNA regulatory network was established. Next, a PPI network of the targeted genes was established to highlight hub genes that might play significant roles in BC progression. Using GEO and TCGA datasets, the lncRNA-miRNA-hub gene network revealed that DGUOK-AS1 was upregulated in BC tissues. Deoxyguanosine kinase (DGUOK) is associated with the phosphorylation of purine deoxyribonucleosides in the mitochondrial matrix. Previous studies revealed that DGUOK mutation or deficiency is the most common cause of mtDNA depletion and is associated with various diseases (28), including neonatal hepatocerebral disease and hepatocellular carcinoma. DGUOK-AS1 is an anti-sense lncRNA of DGUOK, which was recently identified as a tumor-promoter in cervical cancer (29). However, to our knowledge, there are no reports about the expression, or diagnostic and prognostic value of DGUOK-AS1 in BC.

miRNAs are a type of endogenous non-coding RNA with a length between 19 and 25 nt. miRNAs are involved in regulating the development, invasion, and metastasis of various cancers through regulating the expression of oncogenes or tumor suppressors. Many miRNAs play critical roles in the development and progression of BC, including miR-204-5p, miRNA-215-5p, and miRNA-27a. Upregulation of miR-204-5p significantly inhibits the viability, proliferation, and migration capacity of BC cells through regulating various cancer-related pathways and the expression of key cytokines, and high miR-204-5p expression is associated with better survival status of patients with BC (30). miR-215-5p overexpression inhibits the aggressive abilities of BC cells by targeting SOX9 (31), while overexpression of miR-20a-5p promotes the migration and invasion of BC cells through reducing RUNX3 expression (32). We identified miR-497-5p as the key miRNA in the lncRNA-miRNA-hub gene sub-network. miR-497-5p functions as a tumor suppressor to inhibit cell growth and cause cell cycle arrest and is downregulated in several cancer tissues compared with adjacent normal tissues, including ovarian cancer (33), lung cancer (34), lymphoma (35), and BC (36). Metabric database data shows that higher miR-497-5p expression is associated with better prognosis in patients with BC, validating the significance of miR-497-5p in the regulatory network. However, one miRNA can be adsorbed by multiple lncRNAs. In our network, miR-497-5p could be sponged by DGUOK-AS. Li et al. revealed that HOXC13-AS was upregulated in BC tissues compared with the adjacent normal tissues and HOXC13-AS overexpression led to increased cell growth through sponging miR-497-5p (36), making the function of miR-497-5p more complicated and significant.

All of the hub genes with the highest degrees of connectivity have been reported to be closely associated with BC. To explore the potential function of lncRNAs in BC, the hub genes were subjected to functional analyses and several cancer-related GO and KEGG terms were identified. The enriched GO terms, like mitosis and cell cycle, are highly related to carcinogenesis. Among KEGG tumor-related signaling pathways, the p53 signaling pathway contains the largest number of genes and the smallest *P*-value, indicating the potential relationship between DGUOK-AS1 and p53 signaling. As the core of this signaling, p53 functions as a safeguard to maintain genome integrity, which can be regulated by various stimulus or factors. Moreover, dysregulated p53 expression can affect the cell cycle, apoptosis, DNA repair and damage, angiogenesis, and metastasis by modulating downstream target genes (37). In our regulatory network, *CHEK1* and *RRM2* were involved in p53 signaling pathway regulation. After evaluating the association between DGUOK-AS1 and miR-497-5p target hub genes, positive correlations were identified between DGUOK-AS1 and *CHEK1, RRM2*, and *TRIP13*. Using the ENCORI database, we showed that *CHEK1* expression was negatively correlated with the overall survival and high levels of *ANLN* expression was associated with poor disease-free survival in patients with BC. Although no significant association was identified between other hub genes and prognosis, various studies have demonstrated their significant roles in breast cancer (38, 39). *CHEK1* (checkpoint kinase 1) is an evolutionarily conserved serine/threonine protein kinase (40), which plays a significant role in the checkpoint signaling response through phosphorylating crucial cell cycle progression regulators (41). *CHEK1* acts as an oncogene in various cancers, including pancreatic cancer (42), non-small cell lung cancer (43), and ovarian cancer (44). A previous study identified *CHEK1* as a target of miR-497 in hepatocellular carcinoma (45), and *CHEK1* is the target gene of lncRNA CASC9/miR-497 axis in BC. Moreover, *CHEK1* can directly phosphorylate CDC25A, thereby regulating the cell cycle and DNA damage response (46), indicating a potential role of DGUOK-AS1 in tumorigenesis and cancer progression. These results further validate that our DGUOK-AS1-miR-497-5p-centric regulatory network is closely associated with tumorigenesis and the development of BC.

To further explore the biological function and clinical value of DGUOK-AS1 in patients with BC, we evaluated DGUOK-AS1 expression in 40 patients from our center. Our results indicate that DGUOK-AS1 plays a significant role of in BC development. Moreover, 182 BC tissues were collected to analyze the prognostic value of DGUOK-AS1. The Kaplan-Meier survival curve revealed that high DGUOK-AS1 expression might account for poor prognosis in patients with BC. To establish a more accurate prognostic model for patients with BC, univariate, lasso, and multivariate analyses were performed, the results of which further indicate that DGUOK-AS1 could be an independent prognostic indicator for patients with BC. Our novel prognostic nomogram, including both DGUOK-AS1 expression and clinical information, exhibited satisfactory discriminatory abilities and reliable overall survival prediction, indicating promising clinical application prospects. However, the biological function and regulatory mechanism of DGUOK-AS1 in BC remains unclear. Additional research using *in vitro* and *in vivo* experiments are needed to comprehensively elucidate the oncogenic and prognostic mechanism of DGUOK-AS1 in the future.

## Conclusion

Using multiple GEO and TCGA databases, we constructed a ceRNA network that described the potential mechanisms of BC. After analyzing PPI between differentially expressed target genes, hub genes were identified. Subsequently, a more significant and stable DGUOK-AS1-centric subnetwork was constructed. DGUOK-AS1 was highly expressed in BC tissues and was an independent prognostic factor for patients with BC. Moreover, the prognostic nomogram based on DGUOK-AS1 expression displayed favorable discrimination and prediction for prognosis. Bioinformatics analysis also suggested that DGUOK-AS1 might regulate the p53 signaling pathway through sponging miR-497-5p. Our findings indicated that the DGUOK-AS1-related regulatory network could play a significant role in BC and might be a potential prognostic biomarker and therapeutic target for patients with BC.

## Data Availability Statement

Publicly available datasets were analyzed in this study, these can be found in The Cancer Genome Atlas (https://portal.gdc.cancer.gov/); the NCBI Gene Expression Omnibus (GSE60689, GSE112848, GSE119233).

## Ethics Statement

The studies involving human participants were reviewed and approved by Ethical Committee of Qilu Hospital of Shandong University. The patients/participants provided their written informed consent to participate in this study.

## Author Contributions

YLi, TM, and QY conceived and designed the experiments. YLi, YLia, and TM performed the experiments and analyzed the data. YLi and QY wrote the manuscript. YLi and TM contributed to manuscript revision. All authors read and approved the final manuscript.

## Conflict of Interest

The authors declare that the research was conducted in the absence of any commercial or financial relationships that could be construed as a potential conflict of interest.
