# Supplementary material for: Identification of DGUOK-AS1 as a Prognostic Factor in Breast Cancer by Bioinformatics Analysis
Source: Front Oncol. 2020 Jul 17;10:1092. doi: 10.3389/fonc.2020.01092 (PMC7379746; doi:10.3389/fonc.2020.01092)
Supplement: Supplementary file 1 [file Data_Sheet_1.docx]

Supplementary Table I. Associations between patient characteristics and DGUOK-AS1 expression.

| **Variables** | **Cases**  **(n=182) (%)** | **DGUOK-AS1 expression** | | ***P*-value^a^** |
| --- | --- | --- | --- | --- |
|  |  | **Low (n=91)** | **High (n=91)** |  |
| Age | | | | |
| ≤ 50 | 101 (55.49%) | 56 | 45 | 0.136 |
| > 50 | 81 (44.51%) | 35 | 46 |  |
| Grade | | | | |
| 1 | 4 (2.20%) | 2 | 2 | 0.952 |
| 2 | 116 (63.74%) | 59 | 57 |  |
| 3 | 62 (34.07%) | 30 | 32 |  |
| Tumor size (cm) | | | | |
| ≤ 2 | 79 (43.41%) | 43 | 36 | 0.120 |
| 2-5 | 96 (52.75%) | 47 | 49 |  |
| ＞5 | 7 (3.85%) | 1 | 6 |  |
| LN status | | | | |
| Negative | 90 (49.45%) | 42 | 48 | 0.459 |
| Positive | 92 (50.55%) | 49 | 43 |  |
| ER | | | | |
| Negative | 73 (40.11%) | 40 | 33 | 0.364 |
| Positive | 109 (59.89%) | 51 | 58 |  |
| PR | | | | |
| Negative | 75 (41.21%) | 41 | 34 | 0.366 |
| Positive | 107 (58.79%) | 50 | 57 |  |
| HER2 | | | | |
| Negative | 126 (69.23%) | 67 | 59 | 0.261 |
| Positive | 56 (30.77%) | 24 | 32 |  |
| Ki67 | | | | |
| Low | 42 (23.08%) | 24 | 18 | 0.379 |
| High | 140 (76.92%) | 67 | 73 |  |

^a^Chi-square detection.

Supplementary Table II. Primers used for qRT-PCR.

| **Gene** | **Forward (5’-3’)** | **Reverse (5’-3’)** |
| --- | --- | --- |
| DGUOK-AS1 | GACTAACCTCCTCAGAAAAATC | GCAGTTTACATGCAGTTTCT |
| hsa-miR-497-5p | CAGCAGCACACTGTGGTTTGT | CAGTGCGTGTCGTGGAGT |
| Actin | CATGTACGTTGCTATCCAGGC | CTCCTTAATGTCACGCACGAT |
| U6 | CTCGCTTCGGCAGCACA | AACGCTTCACGAATTTGCGT |

Supplementary Table III. The transcript IDs of DElncRNAs in different databases.

| **Ensembl ID** | **NONCODE ID** | **LNCipedia ID** |
| --- | --- | --- |
| ENST00000519762 | NONHSAT216641.1 | MAFA-AS1:2 |
| ENST00000439192 | NONHSAT071644.2 | DGUOK-AS1:5 |
| ENST00000413452 | NONHSAT071645.2 | DGUOK-AS1:6 |
| ENST00000590653 | NONHSAT004288.2 | lnc-LMO4-2:7 |
| ENST00000518014 | NONHSAT104499.2 | CARMN:17 |
| ENST00000545709 | NONHSAT029185.2 | lnc-LEMD3-1:1 |
| ENST00000452919 | NONHSAT087556.2 | CHL1-AS2:1 |
| ENST00000416700 | NONHSAT131648.2 | lnc-ANKRD20A2-1:19 |
| ENST00000443252 | - | lnc-DLK1-35:131 |
| ENST00000420855 | NONHSAT131741.2 | PGM5-AS1:7 |
| ENST00000655915 | - | - |
| ENST00000417887 | NONHSAT131740.2 | PGM5-AS1:6 |
| ENST00000433106 | NONHSAT073793.2 | PGM5P4-AS1:6 |
